# Evolution of chronic hypertensive nephropathies treated with ACE inhibitors on patients in pre-dialysis stage

**Published:** 2010-06-30

**Authors:** Andreea-Cristina Costea, DO Costea, Cristiana David, CN Grasa

**Affiliations:** 1 Nephrology Clinic, “Sf. Ioan” Hospital, Bucharest, Romania; 2 Surgery II Clinic, Constanta District Emergency Hospital, Romania

**Keywords:** chronic renal failure, hypertension, converting enzyme inhibitors (A C E inhibitors)

## Abstract

Arterial hypertension (HT), being the main factor of negative evolution for chronic nephropathies, has imposed a careful adjustment of pharmacological treatment. The widespread use of angiotensin conversion enzyme inhibitors (ACE inhibitors) has brought into attention the side effects of this class of antihypertensive drugs. The study focuses on the clinical and paraclinical evaluation of these elements, by means of detecting variations in serum creatinine, natriuresis and diuresis levels factors. In addition, cardiac cavity measurements have been made and the results have lead to the conclusion that the decision to administer ACE inhibitors has to be well founded, and patients should be closely monitored in order to prevent complications of the primary disease.

## Introduction

Arterial hypertension is an important favoring factor in the evolution of chronic nephropathies towards renal insufficiency. The increase of serum creatinine levels is noticeably faster in hypertensive nephropathy patients, there is early need for implementation of renal substitution therapy and this leads to higher risks of cerebral stroke or acute coronary syndromes. The HT treatment is the most important element through which we can intervene in the evolution of chronic nephropathies towards renal insufficiency, in improving the morbidity and mortality rates and also the patient’s quality of life. The aim value of HT in renal patients is, from our point of view, worth debating [3]. ACE inhibitors will lower arterial BP levels, also improving the sclerosis of all the vascular system, including renal vascularization [4]. The predictable side-effect of ACE inhibitor administrations is the diminishing of primary urine levels and hido-saline retention [5,6] that may raise the BP and this may be more severe with the decrease of the number of functional nephrons (NFA). Reduction of diuresis has another known cause in uremic intoxication. Studies conducted so far have failed to mention or discuss the grade of NFA after treatment with ACE inhibitor. Simultaneous administration of diuretics can lead to electrolyte imbalances or hypovolemia, which, combined with ACE inhibitor administration, become harder to control.

## Materials and methods

The study has been conducted upon the selection of 208 hypertensive patients with chronic renal insufficiency (CRF), who have been admitted and monitored during the period of January 2005 and January 2009 in the Nephrology Clinic of Sf. Ioan Hospital.

We have selected:

1. HT patients with systolic BP (sBP) over 140 mmHG and diastolic BP (dBP) over 90 mmHg over three consecutive determinations at one-week intervals
2. Patients on record who suffer from chronic nephropathy and chronic renal insufficiency (serum creatinine levels between 2 and 10 mg per deciliter.

Structure of the participant group by means of renal pathology:

1. Chronic glomerulopathy – 36% - 75 patients
2. Chronic tubule-interstitial nephropathy – 48% - 99 patients
3. Renal polycystic disease – 8% -16 patients
4. Ischemic nephropathy – 8% - 17 patients

**Table 1:** Distribution of participants by age and sex:

AGE (years)	Share	Male	Female
Sub 30	25	10	15
30-39	42	23	19
40-49	61	29	32
50-59	31	15	16
Over 60	49	21	28
Total	208	98	110

### Methods for evaluating renal function

The patients have been grouped by the treatment they receive. The first lot had initial treatment of HT with ACE inhibitors, and another lot either had late introduction of ACE inhibitor treatment or have had ACE inhibitor treatment withdrawn due to dangerous biochemical parameter developments.

**Assessment protocols:**

- Serum investigations:
  1. Hemoglobin, hematocrit ( for assessment of ACE inhibitor effects on anemia),
  2. urea, creatinine, uric acid,
  3. potassium levels,
  4. natriuresis
- BP monitoring:
  1. daytime BP – self assessment by the patient
  2. daytime BP – during admission periods
  3. Monthly – humoral and clinical assessment in the hospital ambulatory service.
- diuresis monitoring – both in the hospital and at home
- renal ultrasonography
- echocardiography
- electrocardiography

## Results

BP values have a wide variation starting from a sBP of 130 mmHg to 240 mmHg, and dBP of 60 mmHg to 120 mmHg and medium arterial pressure (MAP) of 153.33.

In patients with pre-dialysis there is a prevalence of volume dependent HT. There is a correlation established between the diminishing of NFA and the increasing HT caused by hypervolemia. Even patients with HT of ischemic type start to associate a hypervolemia component, with the BP values becoming dangerously high.

**Diuresis** has been evaluated comparatively on patients who were on ACE inhibitors from the beginning of the study and on those who have had an ACE inhibitor treatment introduced within the progression of the study.

1 0-400 mL/24 h – 4 patients
2 400-1000 mL/24 h – 87 patients
3 1000-2500 mL/24 h – 83 patients
4 over 2500 mL/24 h – 34 patients.

What is worth mentioning is the fact that patients with diuresis levels under 1000ml/24h at the beginning of the study fall into one of two categories:

1 Patients who have had a recent introduction of an ACE inhibitor or an angiotensin receptor blocker (ARB). These patients presented with acute onset of low diuresis
2 Patients with decompensated nephritis, or suffering from intercurrent conditions associated with dehydration, over-imposed on a chronic nephropathy.

The percentage of patients with diuresis over 1000 ml/24h is that of 56% out of 208 subjects.

The distribution of **natriuresis** is varied according to the degree of renal function affected by the subject’s condition. We have measured natriuresis levels only on patients with diuresis over 400 ml/24h.

Patients with chronic nephropathy and secondary HT have pressure natriuresis preexisting to the debut of the HT, and this is a contributing factor to the high BP values; the natriuresis become more and more stabile with the diminishing of the number of active nephrons. This lot of subjects with chronic renal failure shows lower capacity for variation of natriuresis according to sodium ingestion and arterial blood pressure.

What is also worth mentioning, is the paradoxical distribution of natriuresis in chronic tubulopathies with or without low grade renal failure (serum creatinine under 5mg/dl) – 48 patients – which is different from the rest of nephropathies. This is explainable by the affecting of tubular reabsorbing function, with the diminishing of sodium reabsorbtion by the tubular cells with damaged enzyme material, with the result of excessive natriuresis – salt-losing nephropathy.

The next parameter is **proteinuria**, an important prognosis factor for the evolution of chronic nephropathy and also a valuable indicator for the therapy program.

**Table 2:** The distribution of patients by level of proteinuria

Proteinuria range	Under 2 g	2-3,5 g	Over 3,5 g
Number of patients	135	52	21

In the evolution of nephropathies with chronic renal insufficiency, subjects with chronic tubulopathy also develop proteinuria due to segmental and focal glomerural sclerosis, with the value of proteinuria above 2g/24 hours and a relatively rapid evolution towards advanced chronic renal insufficiency.

### Therapeutic interventions during the study and its effects on renal function evolution

Our therapeutic intervention aimed to obtain an optimum control of BP values by recommendation of a low sodium food diet and anti-hypertensive medication, differentiated according to the particularities of the HT, the type of nephropathy, the coexistent conditions and the ongoing evolution of the patients under the administrated treatment: evolution of BP values, diuresis, N retention values.

The number of subjects who were on ACE inhibitors, at the beginning of the study was of 64. We have kept ACE inhibitors for treatment in patients:

-Serum creatinine value under 5.9mg/dl.

-Serum creatinine value rise smaller than 30% of the initial value

-Diuresis over 800 ml/24hrs, with a urinary density of over 1010mg/L

- Patients with no cardiac insufficiency signs -proteinuria of over 2g/24h

There were 45 subjects who met these criteria. For the 19 patients who have decided to take off ACE inhibitor therapy, the motives were:

- Left ventricular insufficiency (LVI) clinically documented both by radiologic and echocardiography methods

- Congestive heart failure phenomena- 6 patients with an initial diuresis of 700-800ml/24hrs (before the introductions of ACE inh.), preexistent cardiac insufficiency of NYHA classes I and II

- Dieresis under 800ml/24hrs or 800-1000ml/24hrs but with a urinary density of 1005mg/L- 6 patients

- Serum creatinine values over 600mg/dl- 10 patients

We mention that all patients have manifested one or more complications secondary to the anti hypertensive treatment, like the operation of left ventricular insufficiency phenomena simultaneous with the diminishing of diuresis under 800ml/24hrs

*The evolution of diuresis in patients taken off ACE inhibitor therapy* – we can observe a rise similar to „kidney pseudo-transplant”, meaning an “addition” of 5-10^5^ to the NFA.

**Diagram 1:**
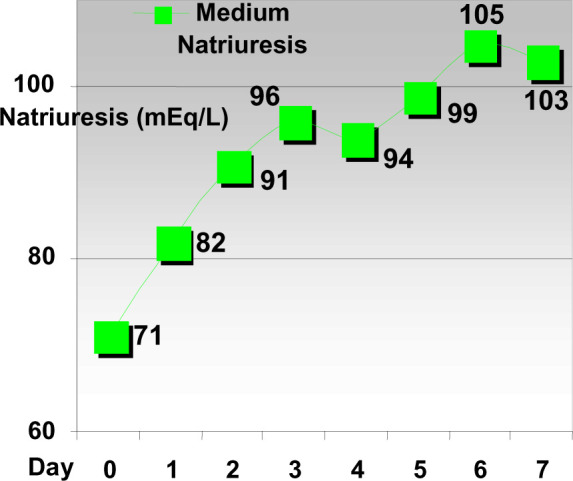
Evolution of natriuresis after discontinuing ACE inhibitor therapy.

**Diagram 2:**
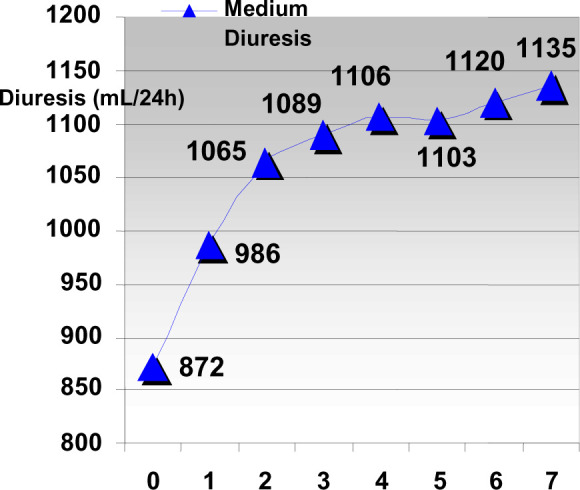
Evolution of diuresis upon discontinuation of ACE inhibitor therapy

The Evolution of natriuresis in patients after discontinuing ACE inhibitor therapy – for the patients to whom we were forced to interrupt ACE inhibitor therapy; we have noticed an important rise in sodium excretion.

The evolution of serum creatinine levels on patients taken off ACE inhibitor therapy because of intercurrent effects–dehydration, hydro-electrolytic imbalance- values drop from the average 4,6 mg/dL – to an average of 3,78 mg/dL.

**Diagram 3:**
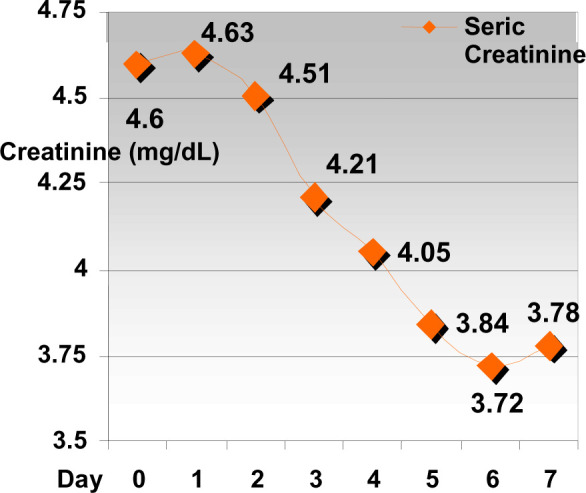
The evolution of serum creatinine levels in patients taken off ACE inhibitor therapy

**Table 3:** The correlation between diuresis-natriuresis and creatinine serum levels in the lot of patients under ACE inhibitor therapy (ACEIT)

	Average Diuresis (mL/24h)	Average Natriuresis (mEq/L)	Average Creatinine Levels (mg/dL)
Without ACEIT initially	872	71	4,60
7 days after stopping ACEIT	1135	103	3,78
p (chitest)	p=0.00074	p=8.8x10^-4^	p=4,1x10^-3^
Diuresis Correlation			
index		0,9687	-0,8059

The study shows a high correlation (p=0.96) between the average natriuresis levels and diuresis, indicating a possible causal relationship, as well as an anti-correlation (p=–0.81) between serum creatinine levels and diuresis. Taking into account the simultaneousness of the administration of ACE inhibitors, we can suspect this treatment as being the common cause of the observed modified parameters.

It is to be noted that glomeruropathies, due to the proliferative membrane lesions with the diminishing of glomerural capillary mass, weaken reactions to ACEIT withdrawal, whilst diuresis spikes were smaller in these subjects.

Subjects we have initiated ACEIT on- 102 patients, presented a serum creatinine level lower than 6 mg/DL. This therapy was initiated in the hospital with monitoring at the 3^rd^ and 7^th^ day and daily diuresis. Those with serum creatinine levels higher than 6mg/DL or important diminishing of diuresis and LVI secondary phenomena have been withdrawn from ACEIT.

The evolution of diuresis on patients we have introduced ACEIT on: The initial average of diuresis was of 1380 ml/24hrs for this lot. The diuresis value dropped with an average of 120 ml/day the first 3 days, than slower with stabilization at 1000ml/day

Evolution of echocardiography parameters:

**Table 4:** The evolution of echocardiography parameters in the glomerulopathy in comparison to other subjects

Diuresis	AS		E/A		TDE		E/Ea	
	6 mo	12 mo	6 mo	12 mo	6 mo	12 mo	6 mo	12 mo
Anuria patients	73,2	88,0	1,9	3,1	134	121	16,5	17,2
Over 1000 ml	38,8	39,8	1,3	1,4	N	N	6,0	6,9

**Diagram 4:**
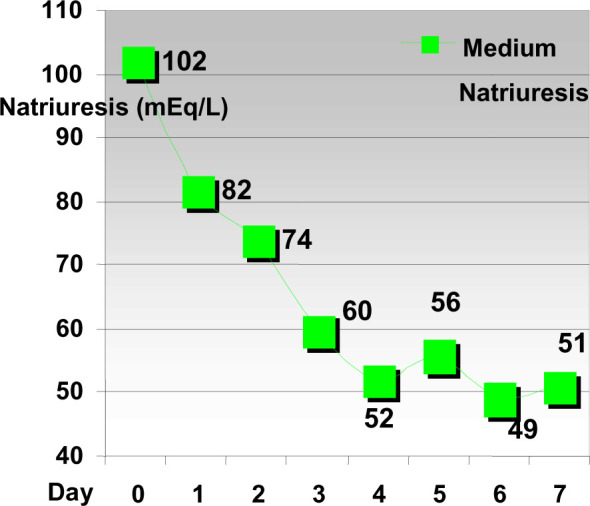
The evolution of Natriuresis on patients with chronic renal insufficiency and on ACEIT

**Diagram 5:**
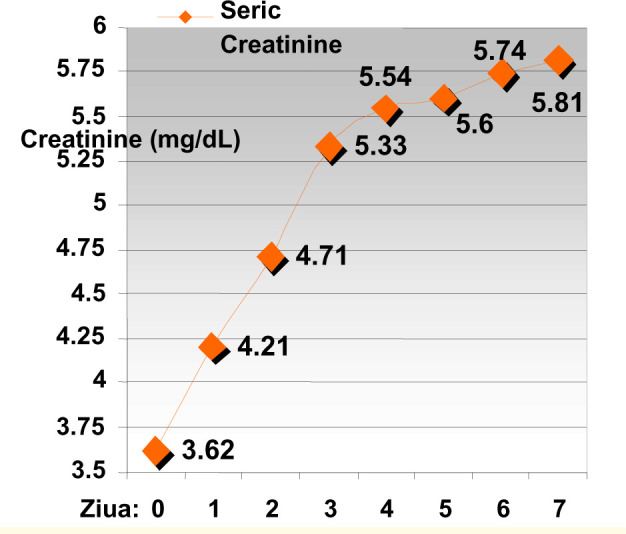
The evolution of Serum creatinine levels on patients with pre-dialysis chronic renal insufficiency on ACEIT

Results show the diminishing of natriuresis respectively with the rise of serum creatinine levels relatively fast in the first 4 days of treatment with ACE inhibitors, after which the values have stabilized at a certain level.

**Table 5:** Correlation between diuresis-nartiuresis-creatinine levels in the ACEIT lot.

	Average diuresis (mL/24h)	Average natriuresis (mEq/L)	Average creatinine (mg/dL)
Without initial ACEIT	1112	102	3,62
3 days after ACEIT	524	60	5,33
7 days after ACEIT	507	51	5,81
p (chitest)	p=0.00053	p=9.0x10^-5^	p=1,1x10^-4^
Diuresis correlation index		0,9808	-0,9764

Since we have high correlations between the average diuresis, natriuresis and serum creatinine levels after the initiation of ACEIT, we can draw the conclusion that there is a causal relationship between their evolutions. Moreover, we can advance the hypothesis that these parameter evolutions are dependent on the initiation of ACEIT in this case.

ACEIT was stopped in 58 subjects:

-Patients with oliguria - exceptions were patients who had very high initial HT levels for whom ACEIT offered a better control of the BP

-Rises in serum creatinine levels with over 30% of the initial value and rises over the value of 6mg/DL

-Patients with LVI phenomena or congestive heart failure aggravated by ACE inhibitors.

## Conclusions for ACE inhibitor administration

The indications for ACEIT must be closely weighed for these patients, paying much attention to each and every case, giving due importance to favoring factors for the complications associated with ACEIT. The benefits of the initiation of this therapy will be quantified in correlation with the possible risks.

-It is to be avoided administering ACEIT to patients with chronic glomeruronephritis although values of BP imposed a potent antihypertensive drug.

-The administration of ACE inhibitors in case of cachexia caused by proteinurias and the resort to “partial non-surgical nephrectomy” is justified.

-In the case of uncontrolled HT, we have administered last resort ACE inhibitors, as we preferred the increase of N retention, and the diminishing of diuresis, this leading to a lower risk of stroke or aggravation of cardiac insufficiency.
